# Termite mound architecture and climate control: a review of X-ray tomography and flow field simulation approaches

**DOI:** 10.1098/rsif.2025.0263

**Published:** 2025-10-22

**Authors:** Nengi Fiona Karibi-Botoye, Guy Theraulaz, Bagus Muljadi, Vasily Demyanov, Kamaljit Singh

**Affiliations:** ^1^ Institute of GeoEnergy Engineering, Heriot-Watt University, Edinburgh EH14 4AS, United Kingdom; ^2^ Centre de Recherches sur la Cognition Animale, Centre de Biologie Intégrative, CNRS, Université de Toulouse III – Paul Sabatier, 31062, Toulouse, France; ^3^ Department of Chemical and Environmental Engineering, University of Nottingham, Nottingham NG7 2RD, United Kingdom

**Keywords:** termite mounds, thermoregulation, ventilation, nest architecture, social insects, X-ray tomography, numerical simulation, flow field simulation, pore-scale modelling

## Abstract

Termite mounds are known for their ability to maintain self-sustained ventilation and thermoregulation irrespective of external climatic conditions. Although there has been extensive interest in this topic, especially for designing energy-efficient buildings, it is still not fully understood how mound properties are controlled. This article reviews established knowledge and identifies gaps in the study of climate control within termite mounds, proposing an interdisciplinary approach that combines X-ray tomography and flow field simulations. Through specific examples, we demonstrate how these methods can deepen our understanding of termite mound structure and its climate-regulating functions.

## 1. Introduction

Termites are regarded as one of the supreme builders within the animal kingdom, skilfully creating structures that provide exceptional control over temperature, humidity and airflow within them [1]. They construct their mounds from materials like saliva, wood, excrement and soil [2–5]. The structural integrity of mounds can persist for several decades to centuries, emphasizing their durability and resilience [4,6,7]. These mounds have captivated biologists, engineers and architects for their ability to regulate heat, humidity and gases without mechanical assistance, even as external climatic conditions change [8,9]. Understanding and potentially replicating the processes underlying temperature regulation and ventilation in termite mounds could advance the fields of architecture and engineering, enabling buildings to meet occupant needs while reducing energy use and carbon emissions [10–13].

Termite mounds vary in shape and size ranging from a few centimetres to several metres with colony size ranging from hundreds to millions [8,14–16]. These structures are crucial to the ecology as they enhance the fertility of the surrounding soil by adding carbon, improving aeration and aiding filtration [17,18]. They also help reduce atmospheric carbon, decompose organic matter and provide habitats for organisms, including *Termitomyces* fungi. Termite mounds mainly result from self-organization processes [19], where the mound structure emerges from individual termites following simple local construction rules [20]. Mound building process is often driven by stigmergic interactions, where termites do not communicate directly with each other but instead respond to external stimuli, such as chemical pheromones and environmental cues like surface curvature [21]. Additionally, vibrational cues play a significant role in mound construction, as the mounds also serve as structures for vibration sensing and signalling [22]. Social factors, such as the movement and density of other termites, further influence the construction patterns within the mounds [23] together with ambient properties like temperature and airflow [16]. While these insights offer valuable understanding, the exact behavioural mechanisms underlying termite mound construction remain a subject of ongoing research.

Mounds can have four distinct zones: the outer mantle, the transition zone, the accumulation zone and the nest [24]. The internal structure of the mound typically consists of a complex network of tunnels, chimneys, surface conduits, royal cells, lateral conduits, ridges and turrets, which vary based on the species of termites. The term ‘mound’ is mostly used interchangeably with ‘nest’. In this article, we use the term *mound* to describe the large epigeal part, whereas the interior of the mound where the fungus (if present) and termite queen reside is referred to as *nest*. The term *channel* refers to millimetre-scale voids in the mound and is sometimes used interchangeably with *chamber* in smaller species, whereas *pore* refers to microscale voids.

Despite recognizing the importance of thermoregulation and ventilation processes in termite mounds, a comprehensive understanding of the underlying mechanisms remains elusive. Our limited understanding can be attributed to the challenges of directly measuring conditions within the mound, particularly in relation to ventilation. While indirect methods such as using tracer gas and condensation on plastic film to study airflow [25–27] have provided valuable insights, they only offer a partial understanding of climate control in termite mounds. Moreover, the diversity of termites with 297 genera and 2951 species [28] each having its own mound design has further hindered our understanding of climate control mechanisms in their mounds. Furthermore, the majority of studies have approached the topic from a biological perspective and have not provided a complete understanding of the climate controlling mechanisms. These limitations highlight the need for new and improved methods of studying termite mounds, especially as the world increasingly prioritizes sustainable solutions.

In this article, we show that X-ray tomography combined with numerical simulations offers insight into how termite mound structures influence internal climate. Although these tools are widely applied in domains such as the oil and gas industry, geo-energy engineering and soil science, their combined use in the context of termite mounds remains underexplored. We present the techniques and workflows required to transition from X-ray tomography of termite mounds to numerical simulation, supported by relevant examples. We also discuss how simulations can be applied across different spatial scales, from microscale to entire mounds. Furthermore, we highlight key limitations of this integrated approach and emphasize the importance of validating simulation results, offering suggestions for how validation can be effectively implemented. In doing so, we show how an integrated approach can help explore the identified knowledge gaps towards bioinspired climate control solutions.

We start by reviewing studies on thermoregulation and ventilation in termite mounds. This is followed by a discussion on the application of X-ray tomography to enhance our understanding of termite mounds, including relevant examples. We also explore how numerical simulations can advance this research. Finally, we summarize key findings highlighting areas that are still unexplored and conclude with insights into potential future directions.

## 2. Thermoregulation in termite mounds

**Figure 1.**
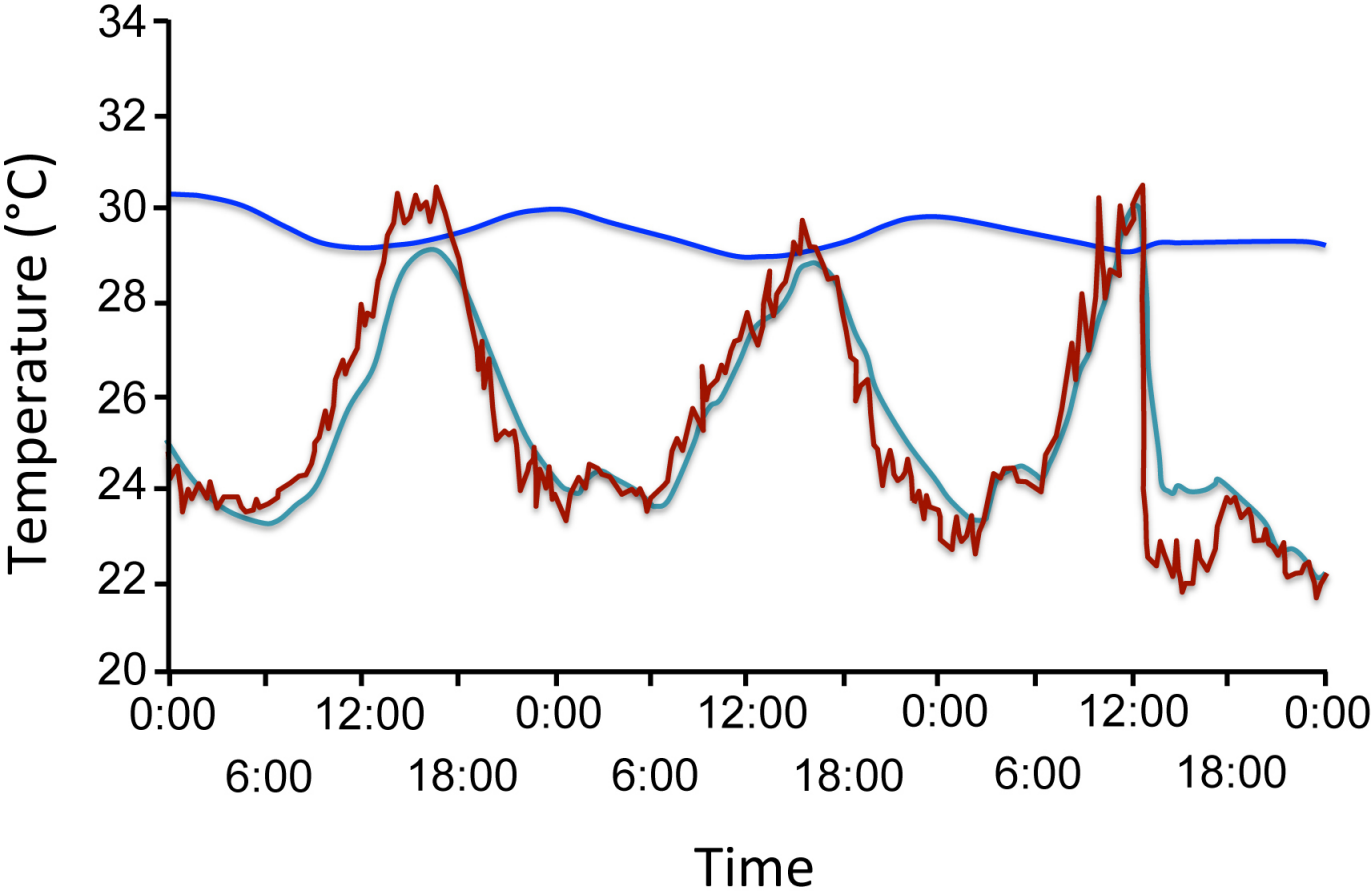
Temperature profile in the mound and ambient temperature over the course of three days. Temperature of air 1 cm above the surface of the mound in red (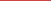), inside the air channels in light blue (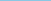) and inside the nest in dark blue (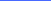) (adapted from Korb & Linsenmair [34] with permission).

Temperature and humidity regulation is essential for termites, especially in species that cultivate fungi, as the termites and fungi require stable environments [29,30]. Termites maintain stable conditions mainly through passive methods like selecting specific mound sites and designs, with minimal reliance on active methods such as clustering for warmth [31]. Several studies have been carried out to investigate the temperature profiles at different locations within the termite mounds [9,26,32–34]. These studies show that temperature varies across mound locations, with internal nest temperatures remaining stable compared with external temperatures, as shown in figure 1. In these studies, the daily temperature variation in nests is minimal (0−4°C) compared with fluctuations in ambient temperature (up to 20°C) [33,34].

The stable internal conditions can vary depending on whether a mound is inhabited, its size and its ventilation type. Inhabited mounds tend to be 1.4–6.1°C warmer than uninhabited mounds due to metabolic heat from termites and fungi [32,34–37] (see table 1). Larger mounds exhibit greater thermal stability compared with smaller mounds [5,32,34] (see table 1). The enhanced temperature stability in large mounds arises from their greater thermal mass as they possess a lower surface area to volume ratio and a higher heat capacity. This characteristic enables them to absorb and retain more heat, gradually releasing it into the external environment, consequently leading to reduced temperature variability.

**Table 1.** Summary of measured parameters in termite mounds.

parameter	value
difference in nest temperature between inhabited and uninhabited mounds	1.4 to 4°C [34]
	3.8 to 6.1°C [32]
	3.3°C [37]
	14.5 to 18.6°F [35]
difference in nest temperature between closed and open ventilation mounds	1.5 to 4°C [33]
difference in thermal stability in the nest of large mounds (LM) and small mounds (SM)	range and coefficient of variation (CV) of LM: 2.5°C (0.028)
	range and CV of SM: 6°C (0.045) [32]
air velocity in the mound (cm s^−1^)	−6 to 6^a^ [38]
	−5 to 5^a^ [9]
humidity values in the mound (%)	70 [26]
	96 to 99 [39]
CO_2_ concentration in the mound (%)	4 to 6 [38]
	0.5 to 6 [9]
	0.01 to 2.4 [27,40]
	1.85^b^ [26]
	0.03 to 0.3 [41]
	0.8 to 2.9 [39]

^a^ negative value is used to indicate downward airflow.

^b^ this value is obtained from P_CO₂_/P_atm_.

Ventilation type also has a critical influence on nest temperatures. For instance, in the closed mounds (mounds with no visible holes) built by *Macrotermes michaelseni*, nest temperatures consistently exceeded those found in open mounds of *Macrotermes subhyalinus* by 1.4–4°C [33]. This difference can potentially be attributed to the absence of visible wall openings in closed mounds, thereby preventing heat loss and minimizing the influx of air in comparison with mounds with open ventilation. Consequently, the adoption of an open mound by some termites might serve as an adaptation that facilitates heat dissipation in regions characterized by high temperatures. This observation suggests that structures primarily designed for gas exchange might also contribute to nest thermoregulation when favourable thermal conditions are available [42].

It is important to highlight that ambient temperature influences the baseline in a mound with higher ambient temperatures leading to higher mound temperatures and vice versa [8,34]. Ambient temperature also influences the mound architecture together with factors such as soil composition [43], solar exposure, humidity, CO_2_ levels and genetics [1,23,44]. It is difficult to determine the primary factor influencing mound architecture, raising the question of whether its construction is driven mainly by environmental conditions, soil composition, or the termites’ genotype.

**Figure 2.**
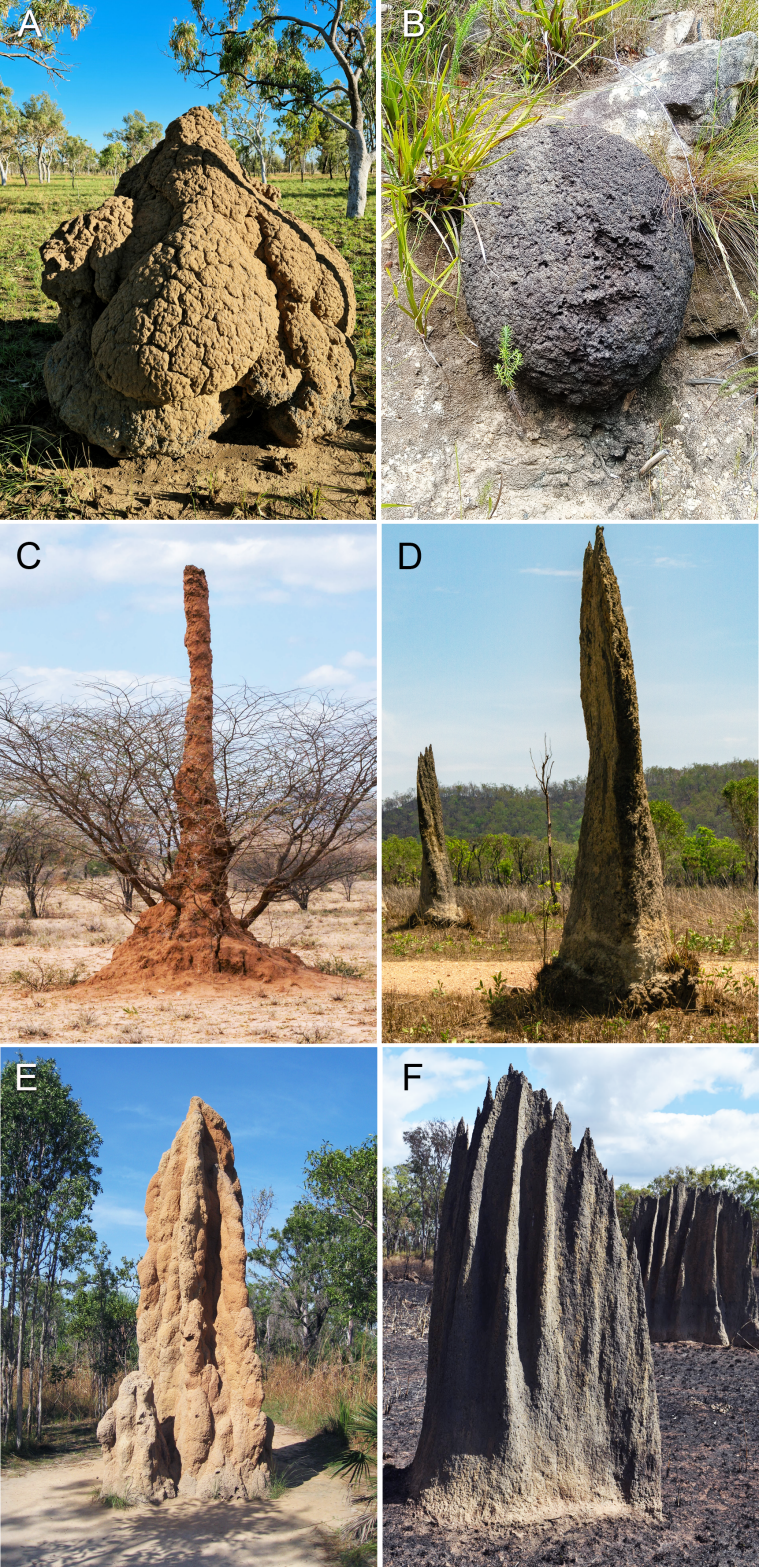
Diversity in termite mound architecture: (A) *Nasutitermes triodiae* in Western Australia, (B) *Amitermes hastatus* in South Africa (photograph by Nicole Loebenberg), (C) *Macrotermes jeanneli* in South Ethiopia (photograph by Rod Waddington), (D) *Amitermes meridionalis* in Northern Territory, Australia (photograph by Peter Szoke), (E) *Nasutitermes triodiae* in Northern Territory, Australia (photograph by John Brew) and (F) *Amitermes laurensis* in Queensland, Australia (photograph by Russell Cumming). All photographs reproduced with permission of the photographers.

Figure 2 shows examples of the diversity of mound architectures built by termites, both across different species and within the same species. Figure 2A,E shows the mounds built by *Nasutitermes triodiae*, with different architectures formed at different stages of construction. These differences in architecture have a direct impact on temperature regulation and ventilation as their internal and external features are different. Even within the same climate, mounds built by the same termite species can exhibit different ventilation mechanisms, adding complexity to the study of termite mounds. Similar architectural diversity is also observed in *Amitermes* mounds across different climates (figure 2B,D and F).

## 3. Ventilation in termite mounds

Termite nests require effective ventilation to exchange CO_2_ produced both by the termites and the fungi they house. Lüscher [39] estimated the oxygen requirement for a mound containing a colony of approximately 2 million termites weighing 20 kg to be approximately 240 l per day. The gas output of large mounds like the *Macrotermes jeanneli* is between 100 000 and 400 000 l of air per day, including 800 and 1500 l of CO_2_ per day [25]. Typically, the oxygen concentration inside a nest is 2% lower than the surrounding air, while carbon dioxide levels are higher by up to 6% [38,45]. Although termite mounds can tolerate more CO_2_ than humans and typically produce more CO_2_ than a single human, these values indicate the importance of effective ventilation in the mound to remove the produced gas.

Ventilation in mounds occurs in two phases [9,46]. The first phase is transport of gases from the underground part of the nest, where metabolic activity takes place (i.e. where the fungus and the brood are located), to the mound surface. In the mounds that do not have an underground part, this first stage consists of transporting the gases from the centre (or core) of the mound to below the outer wall of the mound. Effective connectivity of channels in the mound is crucial in the first phase of ventilation. The connectivity in some mounds can be as high as 98% (see table 2). Furthermore, effective transport within the mound is important, requiring adequate permeability to support fluid flow.

**Table 2.** Structural properties obtained from X-ray tomography of termite mounds.

parameter	mound type	activity/ventilation type	value
microporosity (%)	*Nasutitermes exitiosus*	non-fungus farming/closed	34.1, 32.8 [47]
	*Odontotermes obesus*	fungus farming/closed	6.6 to 8.6 [4]
	*Microcerotermes nervosus* *Macrognathotermes sunteri* *Tumulitermes pastinator*	non-fungus farming/closed non-fungus farming/closed non-fungus farming/closed	23 to 39 [48]
	*Macrotermes michaelseni*	fungus farming/closed	0.71, 1.74 [49]
	*Trinervitermes geminatus*	non-fungus farming/closed	15, 27 [50]
macroporosity (%)	*Coptotermes lacteus* *Nasutitermes exitiosus*	non-fungus farming/closed non-fungus farming/closed	30 to 55 [51]
	*Macrotermes michaelseni*	fungus farming/closed	13.9 [52]
	*Microcerotermes nervosus* *Macrognathotermes sunteri* *Tumulitermes pastinator*	non-fungus farming/closed non-fungus farming/closed non-fungus farming/closed	19 to 36 [48]
channel thickness (mm)	*Trinervitermes geminatus*	non-fungus farming/closed	6.7, 6.8 [50]
	*Macrotermes michaelseni*	fungus farming/closed	3 to 25 [52]
pore size (mm)	*Trinervitermes geminatus*	non-fungus farming/closed	0.073, 0.069 [50]
	*Odontotermes obesus*	fungus farming/closed	0.53 [4]
outer wall thickness (mm)	*Trinervitermes geminatus*	non-fungus farming/closed	14.9, 11.1 [50]
connectivity	*Trinervitermes geminatus*	non-fungus farming/closed	92 to 98% [50]
	*Cubitermes spp*.	non-fungus farming/closed	76 to 96%^a^ [53]
	*Coptotermes lacteus* *Nasutitermes exitiosus*	non-fungus farming/closed non-fungus farming/closed	Euler characteristic^b^ = −100 to 0 [51]
	*Macrotermes michaelseni*	fungus farming/closed	valence^c^ = 3 to 4 [52]
density (kg m^−3^)	*Nasutitermes exitiosus*	non-fungus farming/closed	1052 to 5199 [47]

Mean values are reported and if there are several values measured, it is reported as a range.

^a^ estimated from nodes/total number of chambers.

^b^ estimated from number of isolated objects (N) - number of redundant connections (C) in the pore space + number of completely enclosed cavities (H)

^c^ number of edges connected to each node

Transport within the mound can be driven by both pressure and temperature gradients. The temperature gradient arises from diurnal solar heating, which heats different parts of the mound unevenly, whereas pressure differences can be caused by external winds and turbulence driving airflow. In some mounds, such as those of *Cubitermes* species and magnetic termite mounds like *Amitermes meridionalis*, diffusion primarily facilitates transport within the mound due to its structure [41,54]. These transport mechanisms become even more important as high humidity inside the mound can limit oxygen availability.

The second phase is the transport of gas across the outer wall of the mound to the outer environment. In open mounds, millimetre-sized holes on their outer walls can facilitate mound ventilation. In the absence of holes, ventilation can still occur through the porous microstructure of the outer wall [41,50,55]. In these mounds, ventilation is sensitive and relies on both a well-connected internal network of air channels and a porous and permeable outer wall. Without these connected networks and porous walls, CO_2_ can accumulate within the mound affecting the ventilation process.

Various mechanisms have been proposed to explain how ventilation occurs in termite mounds, ranging from thermosiphon theory and unidirectional ventilation, as seen in tall structures, to tidal effects. The most widely accepted mechanism, confirmed by direct airflow measurements, is solar-powered ventilation, as proposed by King *et al.* [9] and Ocko *et al.* [38]. In their model, solar heating raises the air temperature within surface air channels during the day, causing warm air to rise, thus creating a natural convection effect and pulling cooler air from the nest toward the surface. At night, when the temperature of the surface air channels drops below the nest temperature, there is a reversal in temperature gradients, changing the airflow direction but still facilitating ventilation within the mound. This ventilation mechanism is similar to the external and internal ventilation proposed by Korb & Linsenmair [27].

In every mechanism that powers the mound, the architecture of the mound plays a vital role and strongly influences the air movement and CO_2_ transport. Thus, the structure of the mound must be examined and the properties that optimize CO_2_ transport must be identified to replicate efficient ventilation processes.

## 4. Combined effect of ventilation, thermoregulation and humidity in termite mounds

Termite mounds exhibit a complex interaction of temperature regulation, humidity control and gas exchange, all managed simultaneously within their structure. Studying these processes together is essential. Termites adjust their mound structures in response to environmental factors like humidity, temperature, solar exposure and rainfall. Changing the mound structure in response to one environmental factor may influence other conditions within it. For instance, in cold climates, dome-shaped mounds with thick walls minimize heat loss but can inhibit gas exchange, causing high CO_2_ levels that can lower termite metabolic activity and reduce the internal temperature of the nest, counteracting the mound’s heat retention goal [8,42,56]. Thus, the mound design reflects trade-offs influenced by species, environmental conditions and mound location (above or below ground) [57].

In these interdependent conditions, termites may need to prioritize between thermoregulation and ventilation, as these processes are often related to each other. For instance, some mounds optimize temperature control at the expense of CO_2_ exchange, while others may do the contrary. Completely understanding how these processes influence each other requires detailed experimental and simulation investigations.

## 5. X-ray tomography of termite mounds

Historically, the internal structure of termite mounds has been explored using various visualization techniques, like cement infiltration, mound sectioning and paint tracing, cross-sectional image analysis, planar sectioning and slicing and camera-based scanning [48,55,58], each with its unique strengths and limitations. These techniques do not provide a clear and comprehensive understanding and are often destructive, making the mounds unusable afterwards. Some modern non-invasive techniques, such as electric resistivity tomography (ERT), offer a less intrusive alternative; however, they only provide data on surface-level resistivity values without revealing the full internal structure [49]. This limitation has prompted interest in other imaging methods that could deliver comprehensive, non-destructive insights into mound structures.

X-ray tomography has emerged as a powerful non-destructive tool for visualizing the internal structures of opaque materials [53,59]. Conventional medical computed tomography (CT) scanners can achieve spatial resolutions of approximately 250 µm, which is effective for large-scale samples. With advances in imaging technology, high-resolution X-ray micro-CT and nano-CT now enable more detailed characterization of internal structures [60,61].

Despite its advantages, X-ray tomography faces certain limitations, such as noise, operator variability and imaging artefacts, though effective filtering and image processing techniques can address these challenges. Beyond its application to general imaging, X-ray tomography has shown promise in laboratory settings, particularly in soil sciences, geo-energy engineering and the petroleum industry for analysing fluid saturations and characterizing porous media [62,63]. The insights from such analysis are potentially applicable to termite mounds, where understanding internal structures could aid in the study of thermoregulation and ventilation dynamics.

### 5.1. Millimetre-scale and microscale termite mound tomography

**Figure 3.**
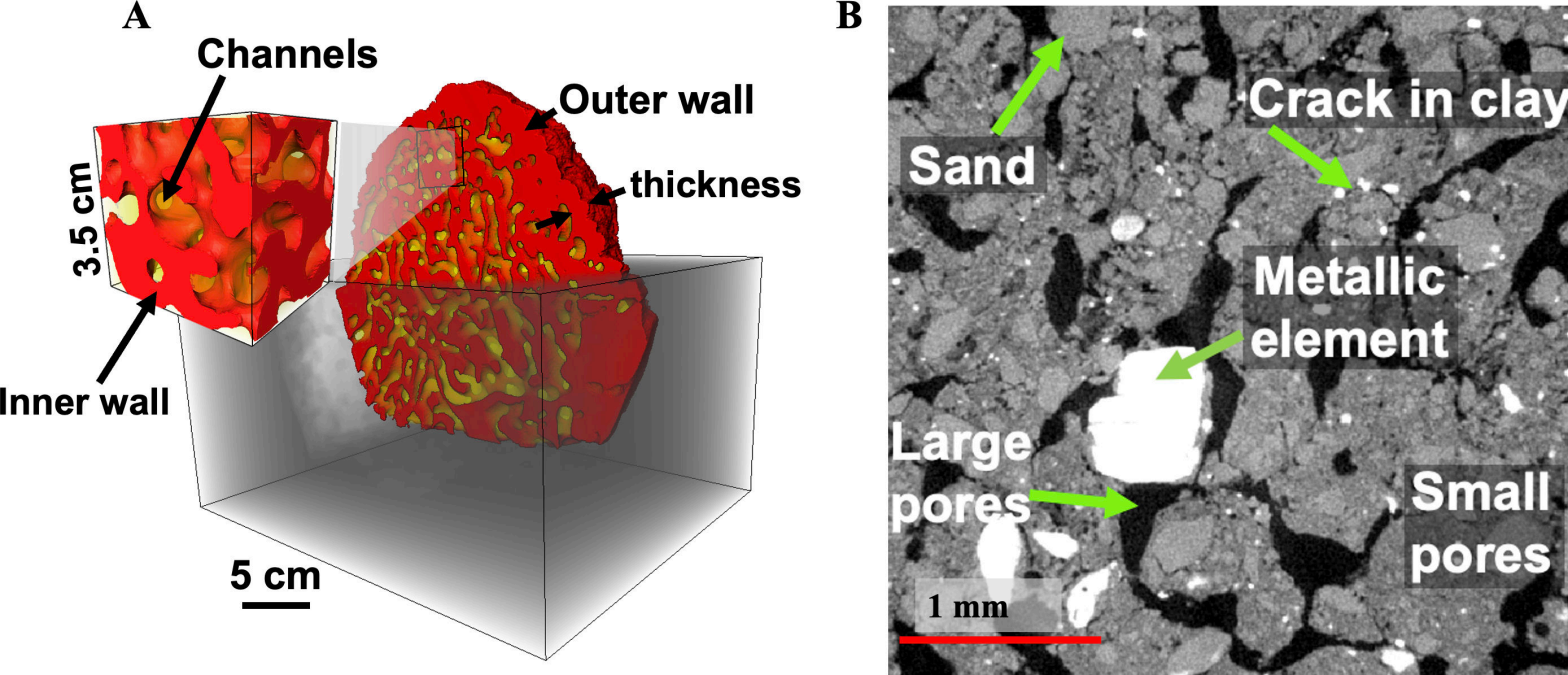
X-ray tomography of a termite mound from the *Trinervitermes geminatus* species. (A) Three-dimensional image showing channels, inner walls and outer walls and (B) a vertical cross-section of an X-ray micro-tomography scan showing micropores of different sizes, metallic elements sand and cracks (adapted from Singh *et al.* [50]).

**Figure 4.**
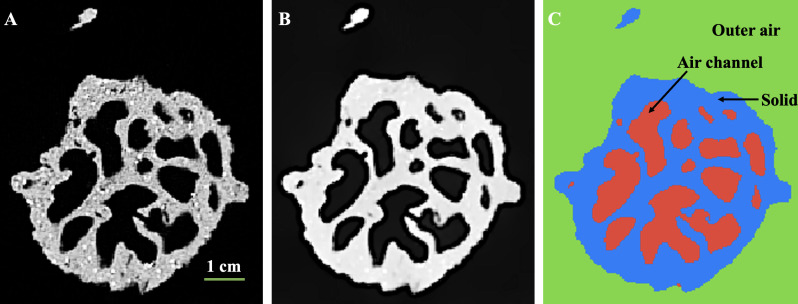
Image processing and segmentation. (A) original grey-scale image, (B) filtered image using non-local means filter and (C) segmented image showing air channel, solid and outer air.

**Figure 5.**
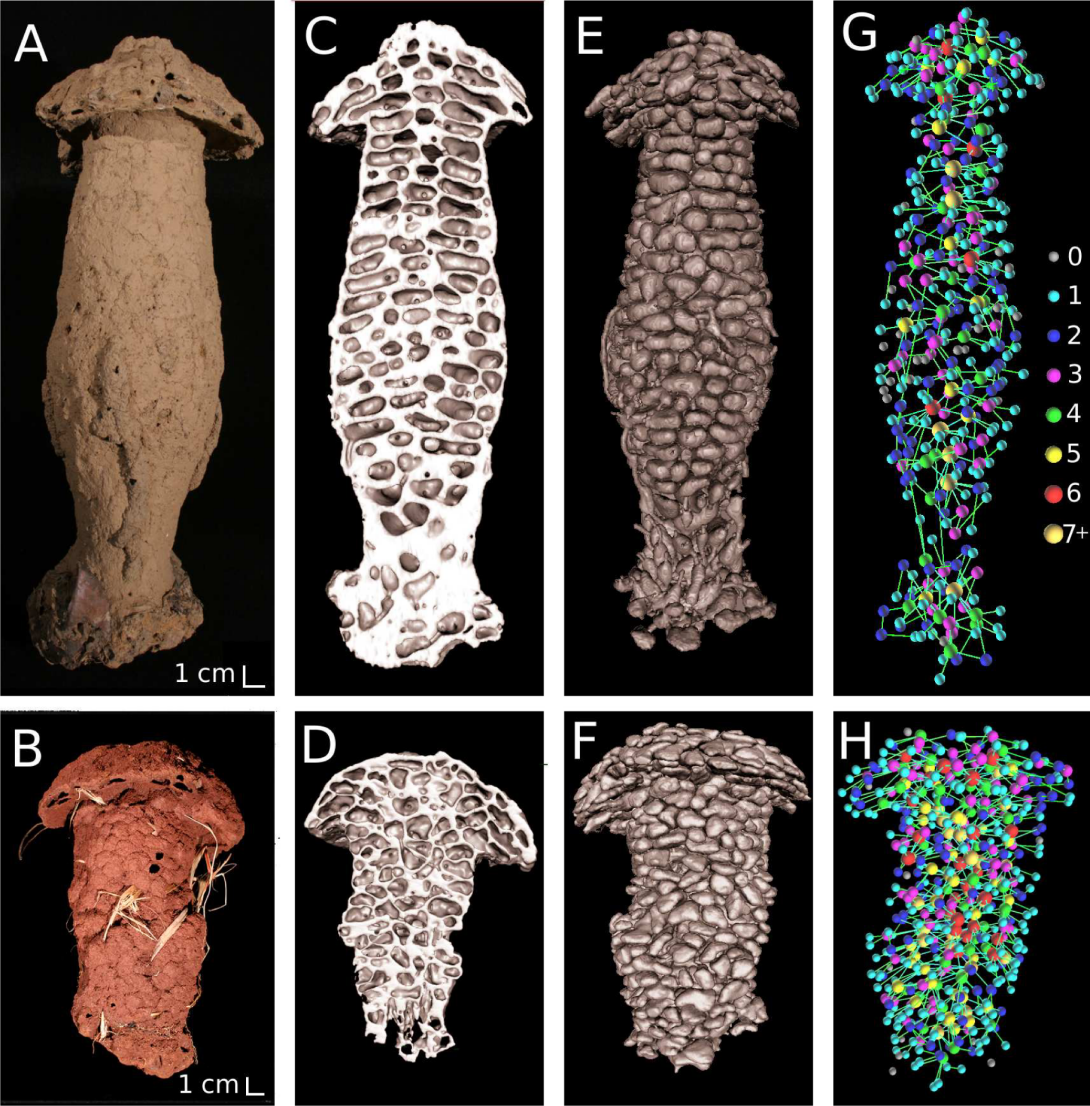
Analysis of *Cubitermes* mound from forest (top row) and savannah (bottom row). (A) and (B) show images of the mounds, (C) and (D) show topographical slice of the mounds, (E) and (F) show virtual ‘cast’ displaying chambers and galleries (virtual cast here means digitally simulating filling the mound with a material like gypsum, allowing it to set and removing the solid parts of mounds revealing the chambers and galleries), and (G) and (H) show network of chambers and galleries in the mounds. The colour of the chambers in (G) and (H) reflects their degree of connectivity (number of corridors connected to a chamber) (Perna *et al*. [53] with permission).

**Figure 6.**
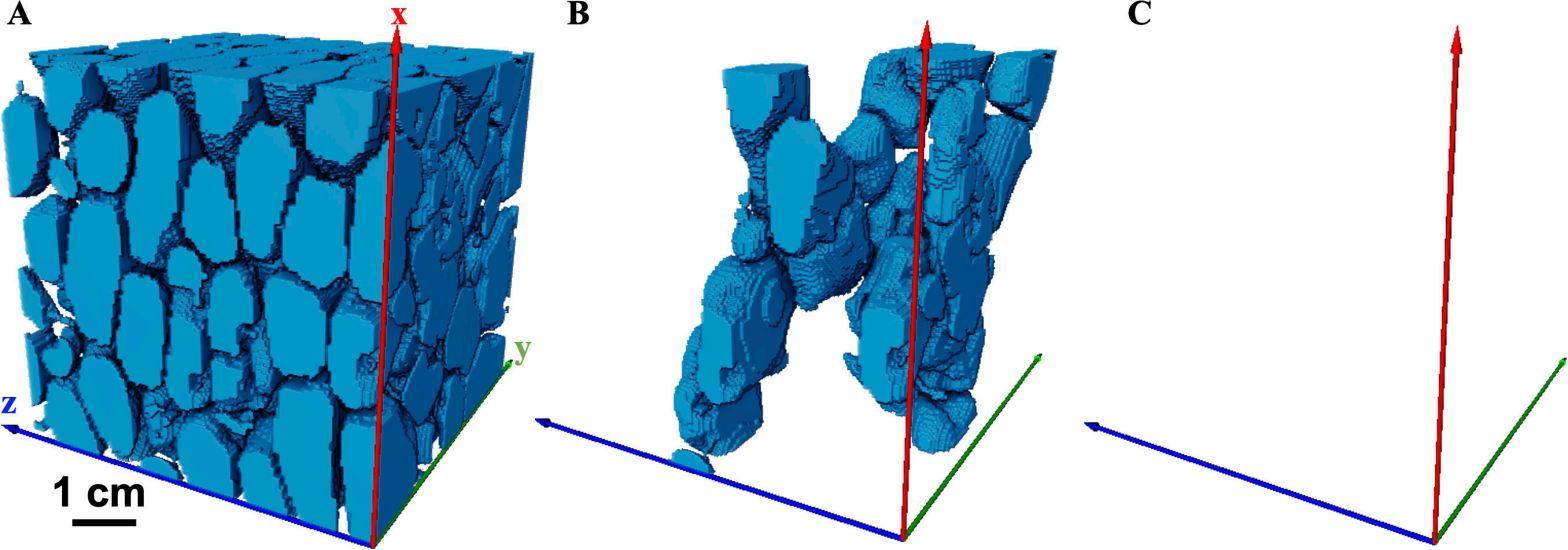
Connectivity in a subset of *Thoracotermes* mound. (A) Virtual ‘cast’ showing chambers and galleries in the mound. (B) Connected chambers and galleries in the *x*-direction, where the connectivity is 44% of the total void space. (C) connected chambers and galleries in *z* direction with no connectivity. In this mound, there would be preferential fluid flow in the *x*-direction. This mound was occupied by a smaller species than *Thoracotermes*, who reduced the size of its galleries. This may explain the connectivity being lower than the typical values for this species.

**Figure 7.**
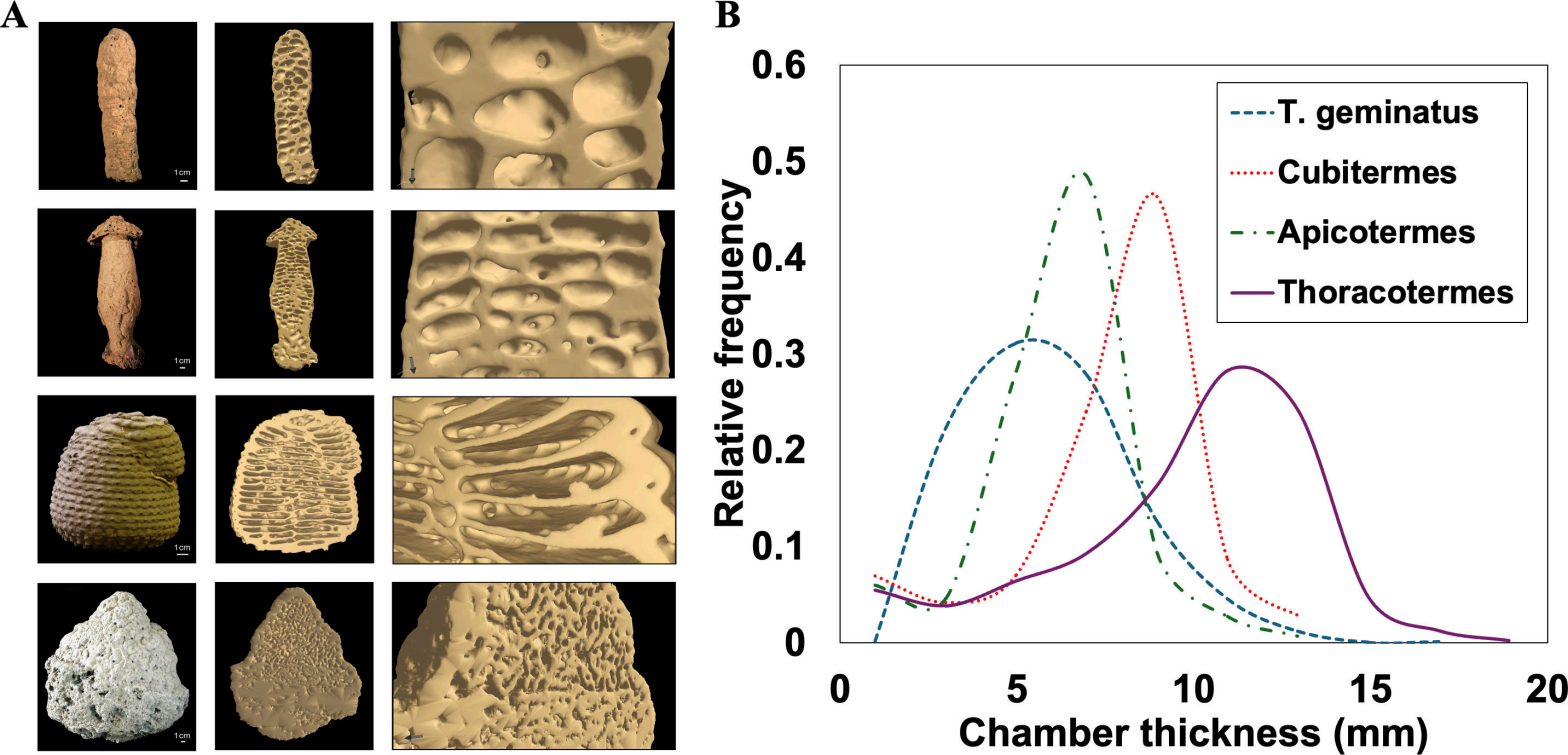
Different termite mounds and their corresponding chamber thickness distribution. (A) Images of mounds. From top to bottom: *Thoracotermes, Cubitermes, Apicotermes* and *Trinervitermes (T.) geminatus* mounds. The white scale bar shown on the image on the left corresponds to 1 cm. The right column shows zoomed-in images of the chambers and galleries of the respective mounds. (B) chamber thickness distribution of mounds in (A). *Cubitermes* and *Apicotermes* exhibit similar distributions, while *T. geminatus* and *Thoracotermes* display a wider range of chamber thicknesses, with *Thoracotermes* tending toward higher values.

Conventional X-ray tomography enables us to visualize an extensive volume of the mound and in some instances the entire termite mound, capturing large-scale structural features. This imaging approach, which dates back to 1956 by Desneux [64], aids in understanding the correlation between large-scale features, such as chamber and outer wall thickness and the mound functionality. Figures 3–7 show examples of X-ray tomography at millimetre scale, highlighting structural patterns of termite mounds from various termite species and capturing a clear distinction between solid parts of the mound and the intricate channels within. For mounds larger than the field of view, X-ray tomography can be conducted on subvolumes obtained from the mound, which can be merged to construct the complete mound.

X-ray micro-tomography offers a closer look at fine-scale structural details within termite mounds, achieving a voxel size of 1 µm or finer [50]. This level of detail enables the identification of small features such as micropores, metallic elements and cracks (figure 3B), which cannot be resolved fully in millimetre-scale tomography. Although X-ray micro-tomography has found extensive application in various fields [65–68], its use in termite mound studies remains limited, with only a few notable studies, including work by Singh *et al*. [50] and Oberst *et al*. [47,51]. However, the smaller field of view in X-ray micro-tomography restricts it to localized sections of the mound. The complementary nature of millimetre-scale and microscale tomography suggests that combining both scales is likely to provide a comprehensive view of termite mound architecture, capturing large-scale features alongside minute details.

Image processing techniques are essential for enhancing and analysing millimetre-scale and microscale X-ray tomography mound images. Filtering methods, such as non-local means filtering, reduce noise in the images without blurring the edges or material boundaries, thereby improving the quality of the images [69,70] as shown in figure 4A,B. Once filtered, the grey-scale image is segmented into distinct components, e.g. figure 4C, where the millimetre-scale image is segmented into solid mound walls and air channels. The microscale images can be segmented into more distinct components, such as individual pores, grains and metallic elements, as shown in figure 3B. Once the segmentation is complete, these images can be used for further analysis to assess structural properties of the mound. Specialized open-access software, such as Fiji, Dragonfly and other commercial options, can be used for image processing and analysis. Some of the key parameters that can be obtained from this image analysis include pore size distribution, wall thickness, chamber size and shape, and chamber or pore connectivity. Details about these software tools are shown in table 3.

**Table 3.** Useful open-access software for image processing and simulations.

name of software	use	source
Fiji	image processing and machine learning tool for segmenting three-dimensional images	https://imagej.net/software/fiji/downloads
Dragonfly	image processing, segmentation and analysis of three-dimensional images	https://www.theobjects.com/dragonfly/index.html
OpenFoam	open-source tool for computational fluid dynamics (CFD)	https://www.openfoam.com/current-release
GeoChemFoam	open-source tool based on OpenFoam CFD toolbox specifically designed for porous media flow processes	https://github.com/GeoChemFoam
ParaView	open-source visualization tool	https://www.paraview.org/download/
Drishti	open-source visualization tool	https://github.com/nci/drishti

### 5.2. Application of X-ray tomography in termite mounds

X-ray tomography has been used in several studies to characterize and quantify the internal structure of termite mounds. In this section, we explore the application of CT imaging in termite mounds, highlighting insights from previous studies, our own analysis and how these techniques can be applied more efficiently in the future.

X-ray tomography can be used to map the network of chambers (air spaces for movement) and galleries (air tunnels connecting chambers) within a mound, as demonstrated by Perna *et al*. [23,53]. This image analysis can provide information on the connectivity of chambers, a key structural element that is essential for various processes such as communication and defence [53]. Additionally, the connectivity analysis can help in understanding airflow in different directions (figure 6). This is especially important in solar-powered ventilation, as even with a thermal gradient present in the mound, a lack of connectivity to maintain continuous airflow could still lead to CO_2_ build-up. The spatial arrangement of chambers and galleries, along with their connectivity across different mound types allows for comparisons between species in different environments. This can reveal how mound structures adapt to local environmental factors. Typical connectivity values observed in different mounds are shown in table 2.

X-ray tomography can also be used to determine the porosity and volume of termite mounds, which are essential structural properties, offering insights into termite behaviour and resource allocation. This technique has been applied in several notable studies, including those by Hervier *et al*. [71], Nauer *et al*. [48], Singh *et al.* [50], Zachariah *et al*. [4] Andréen & Soar [52] and Van Thuyne *et al.* [49]. Porosity also affects the mound’s ability to regulate moisture, air and heat flow, making it a critical factor in climate control. Additionally, porosity, which is inversely correlated to the density of the mound, influences its structural stability. This relationship is evident in the mound’s structure as denser inner walls of the mounds, resembling the architecture of trabecular bone, provide load-bearing support, while the less dense and porous outer walls aid in ventilation and temperature regulation [47]. It is important to note that porosity can be measured at various scales. At the millimetre scale/macroscale, referred to as macroporosity, it represents the ratio of chambers, galleries and conduits to the total volume. At the micro-scale, microporosity refers to the volume fraction of micropores within the mound walls [48] (see table 2). Experimentally measured porosity of the mound walls ranges from 37 to 47% [9,72], comparable to the values in table 2.

In addition to porosity, X-ray tomography reveals pore size distribution, chamber thickness and shape across different mound types. For example, chamber thickness in mounds from *Trinervitermes geminatus*, *Cubitermes*, *Apicotermes* and *Thoracotermes* was compared, revealing that *Thoracotermes* mounds had notably thicker chambers than the other mounds as shown in figure 7. Elongated and thinner chambers as found in *Apicotermes* mounds have been linked to higher permeability in the inner section of the mound (from personal observation). Within the same mound, channel (pore) sizes can be compared across different sections as demonstrated by Zachariah *et al.* [4]. This image analysis can help us link structural differences across different parts of the mound to their potential functions. The porosity and channel (pore) size distribution can also be linked to its connectivity. Larger and well-connected channels (pores) facilitate better airflow, while smaller or isolated channels (pores) may restrict ventilation. Additionally, the shape of channel (pores) may also play a role in flow, as elongated or irregularly shaped channels (pores) could influence directional airflow and drainage in the mound.

X-ray tomography has also been applied in unconventional areas, such as termite pest management, where it enables non-destructive monitoring of wood consumption by termites [73,74]. In the future, image analysis in termite mounds can be extended to include other structural properties such as surface area and surface-to-volume ratio, contributing to a more comprehensive understanding of termite mound architecture. Greater emphasis should also be placed on submillimetre X-ray tomography of the mounds such as those studied by Oberst *et al*. [47] and Singh *et al.* [50] as small-scale features play a significant role in processes like gas exchange, drainage, thermal conductivity and mechanical stability.

Beyond structural analysis, X-ray tomography can be used to study dynamic processes in termite mounds, as demonstrated by Singh *et al.* [50], who monitored fluid distribution to assess how mound structure affects drainage. This approach can be extended to other termite species and to monitor dynamic processes like CO_2_ transport in the mound.

X-ray tomography can also be extended to monitor structural changes occurring in termite mounds over time. This is particularly important as termite mounds are not static structures but are continuously remodelled by termites [38,53]. These changes occur in response to ambient conditions such as temperature. Experiments have shown that altering the ambient temperature of a typical forest environment to mimic savannah temperatures can lead to the reconstruction of the mound and vice versa. In cases where reconstruction cannot be performed quickly, the mound colony may die off [75]. Regions within the mounds that are no longer needed may be closed off, and other structures may need to be reopened. For example, during the rainy season, when the walls of mounds become wet, complex egress tunnels emerge on the mound surface to promote ventilation [52,55]. Analysing such dynamic changes with X-ray tomography would help us link internal structural changes to specific seasonal conditions and climate.

## 6. Numerical simulations on three-dimensional structures of termite mounds

Numerical simulation integrated with X-ray tomography provides a powerful approach for analysing intricate processes within termite mounds in a controlled, efficient and cost-effective way. The fluid flow behaviour, such as velocity, pressure and turbulence, can be analysed alongside key mound properties like permeability, effective diffusivity, tortuosity, dispersion and effective thermal conductivity [55].

For such numerical simulations, segmented X-ray tomography images of mounds are discretized. Discretization involves converting a continuous function or variable into a discrete one. This is the first step in numerical simulation analysis, as it makes the functions and variables manageable for computer processing [76]. There are three types of discretization that can be performed: spatial, equation and temporal [55]. Spatial discretization divides the computational domain, in this case, the mound structure, into subdomains, creating a mesh. A mesh consists of triangles, line segments, points and their *n*-dimensional counterparts [76]. Meshes can be structured, unstructured or hybrid (a combination of structured and unstructured meshes). Structured meshes are composed of hexahedral elements built on a coordinate system and are commonly used for simple geometries like cubes [55]. Unstructured meshes are composed of tetrahedral elements and are better suited for complex geometries, but they can be more computationally expensive.

**Figure 8.**
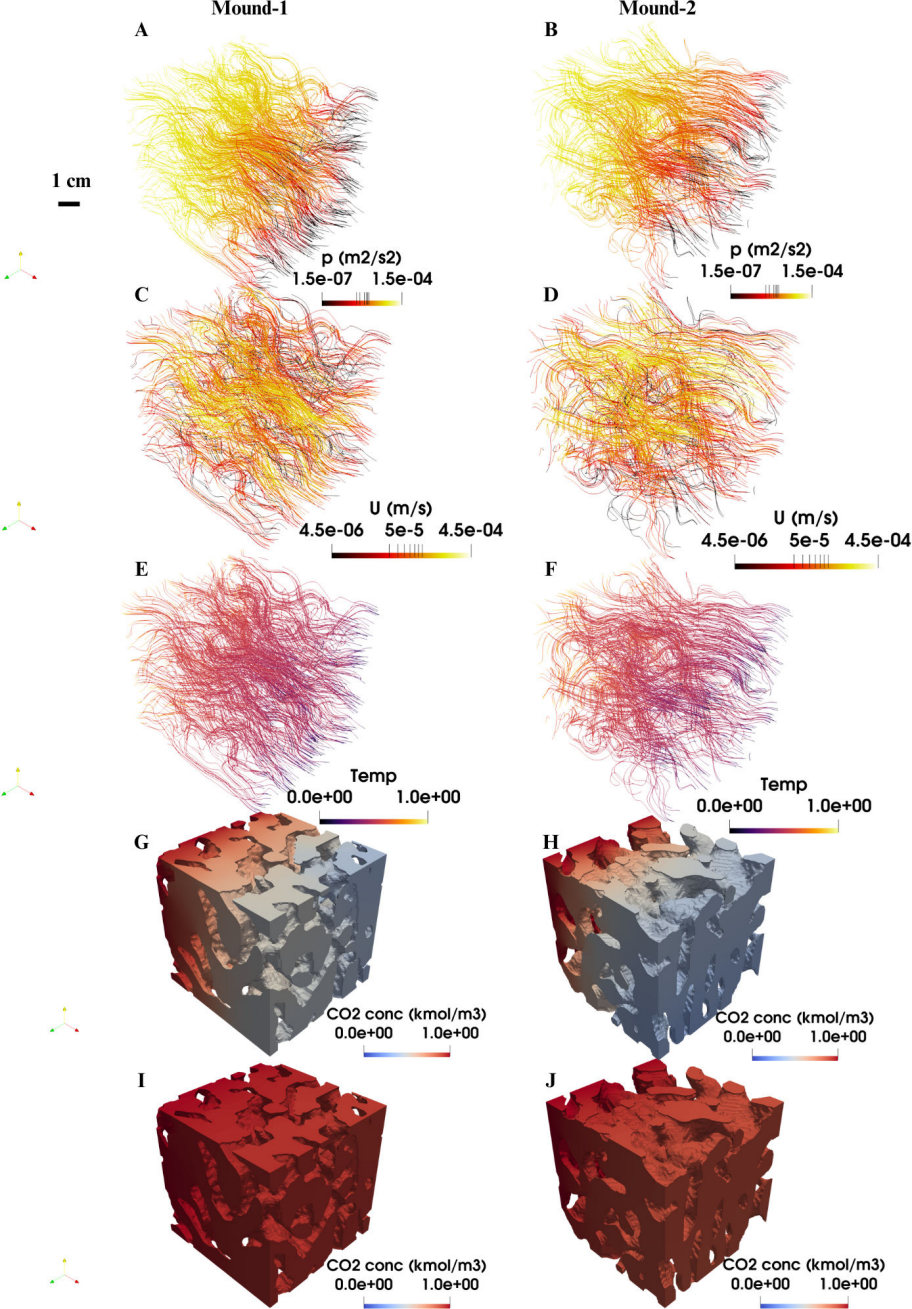
Flow fields, heat and CO_2_ transport at millimetre scale in two mounds (mounds 1 and 2) of *Trinervitermes geminatus* species. Mounds 1 and Mound 2 are the subsets of different mounds from Guinea. (A) and (B) show pressure fields expressed as kinematic pressure (pressure divided by density) from millimetre-scale flow field simulations using Darcy–Brinkman–Stokes equation, applying a flow rate of 1 × 10^−7^ m^3^ s^−1^ at the inlet, zero gradient at the outlet and zero velocity at the walls. (C) and (D) show velocity fields in the chambers of the mounds. (E) and (F) show dimensionless temperature profile in the chambers of the mounds. (G) and (H) show CO_2_ transport through mounds 1 and 2 at 90 s. (I) 100% CO_2_ saturation in mound 1 at 320 s. (J) 90% CO_2_ saturation in mound 2 at 320 s.

**Figure 9.**
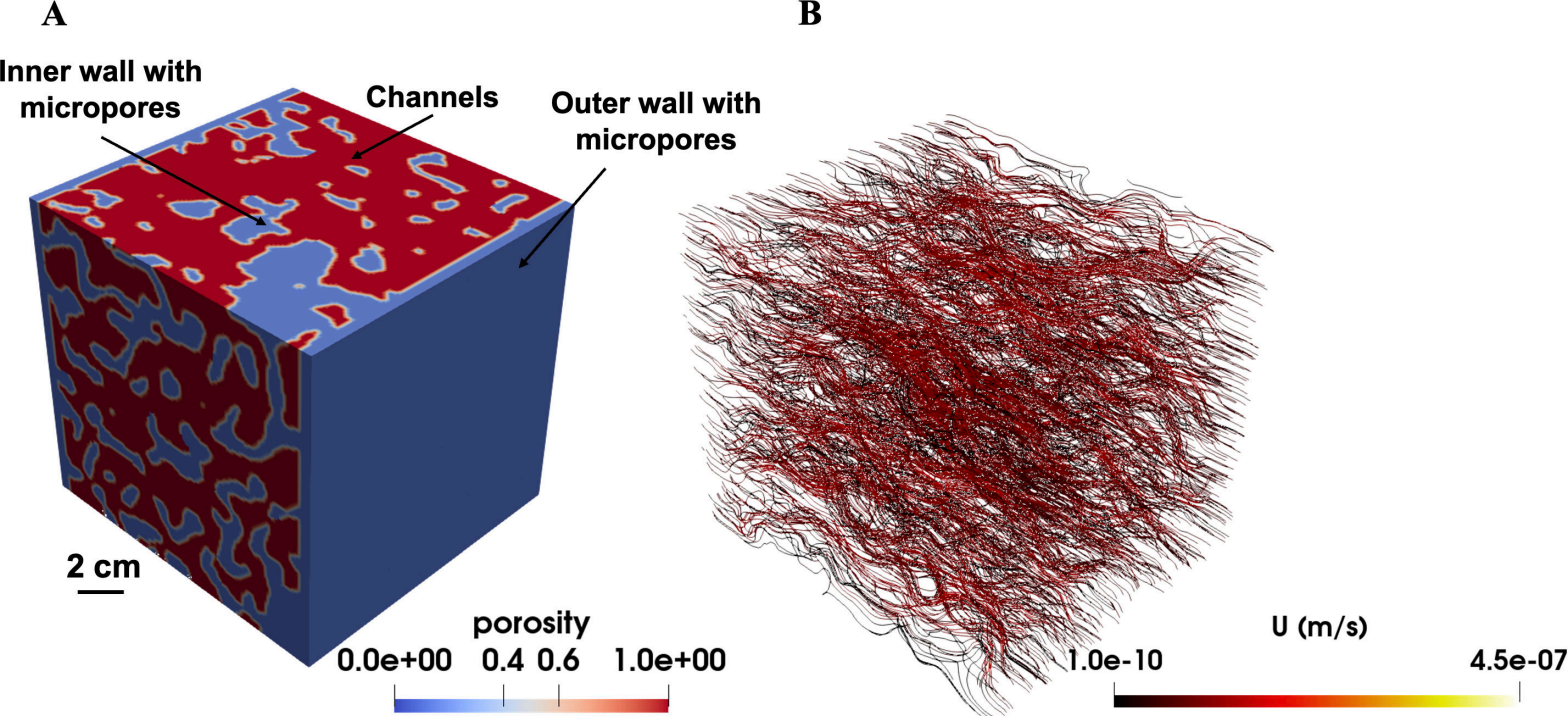
Multiscale simulation in termite mound using Darcy–Brinkman–Stokes equation. (A) three-dimensional structure of a mound showing channels, and microporous walls with porosity of 15% and permeability of 2 × 10^−12^ m^2^. (B) velocity field in the mound.

The mesh can then be refined in regions of interest to improve its resolution. However, it is important to ensure mesh independence, where changes in mesh resolution do not affect the accuracy of the numerical results [55]. Mesh independence can be determined by carrying out simulations at different mesh resolutions and comparing the results to ensure there is minimal variation. For tomographic mound images, a structured grid where each voxel is converted into a numerical grid cell of the same size will be sufficient with local refinement applied as needed. This approach was used to generate the mesh used in figures 8 and 9, as well as those from Singh *et al*. [50]. Additionally, channel (pore) networks can be extracted from these tomographic images to analyse flow [65]. However, this method tends to be less accurate for termite mounds at the millimetre scale.

Equation discretization transforms partial differential equations (PDEs), such as the Darcy–Brinkman–Stokes (DBS), Navier–Stokes and heat equations into numerical forms solvable by computers. Methods such as the finite difference method (FDM), finite element method (FEM) and finite volume method (FVM) can be used for this discretization. FDM is relatively easy to implement and can handle structured grids. However, it cannot handle complex geometries effectively [77]. FVM and FEM, on the other hand, can handle both structured and unstructured meshes. FEM does better with more complex geometries but requires more computational power than FVM. FEM has been used to investigate the role of the external environment on diversity of mound structure [56,78] and is well suited for this type of application, where different mound structures are generated. FVM involves solving equations over finite subdomains known as control volumes. It is the prevalent discretization method in a computational fluid dynamics (CFD) toolbox such as OpenFoam and GeoChemFoam (see table 3). It has been used by Abou-Houly [55] and Singh *et al*. [50] in termite mounds studies. FVM was used to discretize the governing equations for the simulations shown in figures 8 and 9. Within this method, the divergence operator ∇·(...) was approximated using Gauss’s divergence theorem, with face-centred values interpolated through a second-order accurate linear scheme. Similarly, the Laplacian term ∇²(...) was discretized using a Gauss linear uncorrected scheme. These methods are well tested, accurate and stable [79,80]. The choice of discretization method depends on the process being analysed and the computing resources available. However, for characterizing fluid flow in large mounds, FVM is a reliable option.

The third type of discretization is temporal discretization, where continuous time is divided into discrete time steps. For steady-state flow, where fluid velocity, temperature and CO_2_ concentration remain constant over time, this discretization is not part of the mathematical model. However, in practice, steady-state solutions can be obtained either by solving the steady equations directly, for example, Navier–Stokes equation can be solved using semi-implicit method for pressure-linked (SIMPLE) algorithm, or by running a transient solver like pressure implicit with splitting operators (PISO) until the solution converges and no longer changes with time. When using PISO, time must be discretized, since the solver integrates the equations over time, even if the ultimate goal is a steady solution. In contrast, for truly transient flows, temporal discretization must be included to capture the evolution of flow, CO_2_ and thermal properties over time. An explicit method, which is easier to implement but less stable, can be used, as can an implicit method, which offers greater stability. For the CO_2_ transport over time within the *T. geminatus* mound (see figure 8G–J), an implicit first-order accurate Euler scheme was used.

Governing equations are solved within these grid cells iteratively under specified boundary conditions until they converge. These boundary conditions model how the mound interacts with the surrounding environment, the soil beneath it and its internal structures. A fixed value boundary condition can be applied to define the exact airflow velocity, temperature and CO₂ at the inlets and outlets, ideally using measured values from different climatic and environmental conditions to validate these simulations. For example, wind speed, solar irradiation and ambient temperature values typical of Namibia were used in airflow and heat simulations at the inlet of the mound [56]. Alternatively, a fixed gradient boundary condition can be applied for flow simulations, corresponding to the pressure gradient measured in the field [55], or a CO₂ concentration gradient across the mound, scaled relative to atmospheric CO₂ concentration [50]. Time-dependent boundary conditions are particularly useful for heat simulations as they account for diurnal temperature changes at the mound walls, as demonstrated by Ocko *et al*. [16]. Other boundary conditions, such as zero heat flux to model insulation layers and cyclic boundaries, are also applicable.

Convergence in these simulations is reached when the difference between the left and right sides of the equations becomes very small and shows minimal variation between iterations. For instance, in the steady-state flow simulations in figure 8A–D convergence was achieved when the residual (the numerical error remaining after each iteration) was less than or equal to 10⁻⁶. This value is chosen because it is small enough to ensure accuracy without being computationally wasteful; a similar residual threshold was used by Abou-Houly [55]. These equations can be solved using various numerical methods. For example, in the case of the Navier–Stokes equation, algorithms such as SIMPLE or PISO are commonly applied for pressure and velocity coupling. These algorithms are available in toolboxes like OpenFoam and GeochemFoam. The solution can be influenced by several factors, including the discretization scheme, boundary conditions and the physical coefficients used. Therefore, it is important to validate the numerical results against laboratory and field data. The results from the simulations can be visualized using software like ParaView (refer to table 3). These techniques have been widely applied in other fields [65,81,82].

The Navier–Stokes equation can be used to solve fluid flow in the mound. Molecular diffusivity can be estimated by solving Fick’s Second Law, and thermal conductivity can be obtained by solving the heat equation (see Singh *et al*. [50] for equations). It may be important to consider including a source term (*q*) in the heat equation to account for heat generated by termite metabolism similar to what was performed by Fagundes *et al.* [56] and Gouttefarde *et al*. [83]. Although the exact impact of this term on the solution is not fully known, when Gouttefarde *et al*. [83] included it in the heat equation, the results showed a good match with field data. Similarly, when transient conditions were applied in simulations, they produced results that closely matched field measurements [83,84]. It may also be worth accounting for convection occurring in the mound, similar to the approach taken by Ocko *et al.* [16] and Hariyanto *et al.* [84] using the advection-diffusion equation (see equation (6.1)). A similar approach could be applied to account for advection of CO₂ in the mound (see equation (6.2)). The permeability of the mound, which indicates how easily fluid can pass through it, can be determined using Darcy’s equation (see Singh *et al*. [50] for the equation). Darcy’s equation is suitable for low channel (pore) velocities, as is typical in termite mounds (see table 1), and for Reynolds numbers less than 1 [85]. In cases where transient flow occurs and the Reynolds number exceeds 1 [52], the Darcy–Forchheimer equation may be more appropriate (see Abou-Houly [55] for equation).


(6.1) $$\frac{\partial ((\phi c_{f}+(1-\phi )c_{s})T)}{\partial t}+\nabla .\left(c_{f}u_{D}T\right)=\;\nabla .\left(k\nabla T\right),$$

(6.2) $$\frac{\partial (\phi C)}{\partial t}+\nabla .\left(u_{D}C\right)=\;\nabla .(\phi D\nabla C),$$

where *T* is temperature, $c_{f}$ and $c_{s}$ are the heat capacities of the fluid and the solid respectively, ϕ is porosity, $u_{D}$ is Darcy velocity (average pore velocity multiplied by porosity), *k* is single field thermal conductivity*, C* is the concentration of the CO_2_ and *D* is the diffusion coefficient*.*

When solving these equations, it is necessary to input parameters representative of the physical system being modelled, in this case, the termite mound. For example, in the Navier–Stokes equation, the density and viscosity of the fluid (air) must be specified. For CO_2_ transport using Fick’s Second Law, the diffusivity of CO_2_ in air must be provided. For the heat equation, thermal conductivity and/or heat capacity and thermal diffusivity of the solid and air phases must be assigned. These values are not always readily available but can be approximated based on the intrinsic thermal properties of the mound soil components. For instance, Fagundes *et al*. [56] assigned a solid-phase conductivity of 0.2 W m^−1^ K^−1^, representing a predominantly clay composition, which is more insulating. However, this may not be representative in all cases, as the sand and clay composition vary significantly across different mounds [50]. The chosen solid conductivity estimates influence the apparent thermal conductivity calculated in the simulation [84]. Thus, this value should be selected carefully to reflect realistic material properties.

With regard to termite mounds, several numerical simulations have been carried out on digital models that mimic mound structure [16,55,56,78]. These simulations have produced comparable results. For example, the heat transfer Péclet number estimated by Singh *et al*. [50] (0−1.43 × 10⁻^3^) falls within the same range estimated by Ocko *et al*. [16] (10⁻⁶ –10⁶). This low Péclet number range is associated with diffusion-dominated conditions as a result of the wind velocity of 0–5 m s^−1^ outside the mound. For higher wind velocities or temperature gradients, the Péclet number may increase and the system could become advection dominated. However, this rarely happens, as heat flow in termite mounds is typically diffusion dominated [56]. Additionally, the heat diffusivity estimated by Gouttefarde *et al.* [83] (2.7–8 × 10⁻⁷ m² s^−1^) closely aligns with values obtained for the Guinea mound in Singh *et al*. [50] (6.83 × 10⁻⁷ m² s^−1^), based on the apparent thermal conductivity of 1.58 W m^−1^ K^−1^ derived from the numerical simulations and the specific heat capacity and density estimated from X-ray diffraction (XRD) analysis. However, the outer wall permeability measured for the *M. michaelseni* mound (2.96–3.25 × 10⁻¹⁰ m²) is significantly higher than that reported for *T*. *geminatus* by Singh *et al*. [50] (0.6–3.5 × 10⁻¹² m²). This difference is probably attributed to the much higher porosity of 60% observed in *M. michaelseni*, compared with a maximum porosity of 27.9% in *T. geminatus,* highlighting the likely influence of species-specific architecture on flow.

Singh *et al.* [50] conducted flow field simulations using X-ray tomography images of termite mounds scanned at a voxel size of 3 μm. These simulations are usually referred to as pore-scale simulations and are valuable for understanding thermal conductivity and permeability at fine scales. Similar numerical simulations can be performed on millimetre-scale CT mound images to assess the influence of larger structural properties of the mound such as its chambers and galleries on fluid flow. Typical velocity and pressure fields obtained from such millimetre-scale simulations are shown in figure 8A–D. Here, the DBS equation (6.3) was solved using the SIMPLE algorithm. At the fluid–solid boundary, a no-slip condition (zero normal and tangential velocity) was applied. A flow rate of 1 ×  10^−7^ m^3^ s⁻^1^ was specified at the inlet, zero gradient at the outlet and zero velocity at the walls. Air was used as the working fluid in all simulations, with properties corresponding to 30°C, including a kinematic viscosity of 1.61  ×  10⁻⁵ m² s⁻^1^ and a density of 1.164  kg m⁻³. These velocity and pressure profiles reveal which chambers contribute to the flow and to what extent. In the *T*. *geminatus* mounds shown in figure 8, the flow distribution varies across different regions, with bright yellow areas indicating higher velocity and red to dark red areas representing lower velocity, where fluid movement slows down. This variation is probably due to differences in chamber size and porosity across the mound.

As discussed in the previous section, a multiscale approach, where termite mounds are scanned at both the millimetre scale and microscale, and the datasets are integrated, can provide a more comprehensive understanding of mound architecture. Techniques such as DBS modelling allow for detailed simulation of fluid flow in both millimetre-scale and microscale regions of the mound. While this approach has not yet been applied to termite mounds, it has been successfully used in other disciplines such as geothermal, human anatomy and in sedimentary rocks [86–88].

The DBS (equation (6.3)) is an extension of the Navier–Stokes equation. It includes a momentum exchange term between the fluid and solid phase, *νK*^−1^*u* which is significant in the solid phase and disappears in the fluid phase [88]. The permeability *K* is obtained from Kozeny–Carman relationship, and it is assumed to be a function of porosity (equation (6.4)). The DBS equation can account for unresolved regions in the mounds, which is common when solving multiscale flow in porous media. An example of multiscale modelling of termite mounds using DBS is shown in figure 9. In this example, flow through the microporous outer walls of the mound is first simulated. The permeability (2  ×  10^−12^ m^2^) and porosity (15%) obtained from the pore-scale simulation are used in the large-scale simulation of the entire mound. The simulation produces the velocity fields shown in figure 9B, along with the permeability of the multiscale mound (2.12  ×  10^−11^ m^2^). A similar multiscale simulation approach can be applied to determine the thermal conductivity, CO₂ diffusivity and other key flow parameters of the entire mound. In this process, microscale simulations provide the microscale mound properties like thermal conductivity, which are then incorporated into the macroscale model of the mound as input parameters.


(6.3) $$\frac{1}{\phi^{2}}\nabla .\left(u⊗u\right)=-\nabla p+\frac{\nu }{\phi }\nabla^{2}u-\nu K^{-1}u,$$

(6.4) $$K^{-1}=\frac{180\;{\left(1-\;\phi \right)}^{2}}{h^{2}\phi^{3}},$$

where *u* is velocity vector, ν is kinematic viscosity, *p* is pressure, *K* is the permeability and *h* is resolution of mesh.

Typical temperature profiles from two *T*. *geminatus* mounds scanned at millimetre scale are shown in figure 8E,F. A dimensionless temperature of 1 was set at the inlet, 0 at the outlet and a zero-gradient condition was applied at the walls. The simulation was run under steady-state conditions (∂T/∂t=0). Air was used as the working fluid, with a thermal conductivity of 0.026  W m^−1^ K^−1^ and a specific heat capacity of 1007  J kg^−1^ K^−1^. The thermal properties of the solid were a conductivity of 6.32  W m^−1^ K^−1^, a density of 3003.16  kg m^−^³ and a specific heat capacity of 767.64  J kg^−1^ K^−1^, estimated based on the mineral composition of the mound material from XRD analysis. The temperature profiles in these mounds are uniform, with low temperatures at convergence and low thermal conductivity (1.68–1.70 W m^−1^ K^−1^), indicating thermal insulation. The similarity in thermal conductivity between these two mounds is probably due to the mounds having similar porosity and being in the same region. This is not the case for mounds from two regions, Senegal and Guinea, with thermal conductivity 4.18 and 1.58 W m^−1^ K^−1^, respectively [50]. The ambient temperature in Kankan, Guinea is cooler than in Nguekokh, Senegal, which may explain why the Guinea mound requires more insulation. This finding highlights the link between mound architecture, environmental conditions and soil composition.

The temperature profiles also show heat movement through the mound, identifying areas that promote heat flow and those that restrict it, offering insights into the influence of structure on heat propagation. For instance, areas with smaller channels (pores) and resultant smaller porosity demonstrated better thermal conductivity than those with larger ones, highlighting the role of channel (pore) size on thermal conductivity [50]. Measuring the time it takes for heat to travel across the mound provides additional insights into its thermal properties.

Flow field simulations can also shed light on how CO_2_ moves through termite mounds, revealing structural features that support efficient CO_2_ transport. We demonstrate how this can be achieved in two mounds (mounds 1 and 2) from the *T*. geminatus species by applying a fixed CO_2_ value of 1 at the inlet of the mound with zero gradient on all other sides (figure 8G–J), under unsteady state condition. The diffusivity of CO₂ in air was set to 1.7 × 10^−5^ m^2^ s^−1^. At 90 s, the higher proportion of blue region in figure 8H compared with figure 8G indicates lower CO_2_ concentration and a shorter CO_2_ transport distance from the inlet in mound 2 than in mound 1. At 320 s, mound 1 is fully saturated with CO_2_ as indicated by the deep red regions everywhere in figure 8I. This is not the case for mound 2, which reaches about 90% saturation at 320 s as indicated by a lighter red tone near the outlet of the mound in figure 8J. This suggests that mound 1’s structure, probably influenced by its higher porosity (0.56 compared with 0.54 in mound 2) enhances CO_2_ transport efficiency. Additionally, mound 1 had a CO_2_ diffusivity of 4.84 × 10^–6^ m^2^ s^−1^ compared with 4.18 × 10^–6^ m^2^ s^−1^ in mound 2. To better represent CO_2_ transport in the mound, future simulations can include high-humidity levels near saturation, reflecting the conditions observed inside the mound.

Flow field simulations also provide insights into permeability variations within termite mounds, revealing how fluid flows through certain pathways while others slow or redirect airflow. This understanding is essential for explaining natural ventilation mechanisms in termite mounds. A comparison of millimetre-scale CT images of *Apicotermes* and *Cubitermes* mounds (shown in figure 7) reveals a significant difference in permeability, with *Apicotermes* mounds exhibiting permeability levels approximately four orders of magnitude greater than those of *Cubitermes* in their inner section. This suggests that the features of the *Apicotermes* mound such as its elongated and parallel chamber geometry, promote better airflow.

These simulations are often simplified using various assumptions to make them computationally manageable and faster to solve. Typically, fluid and heat flow are assumed to be at steady state [50,55]. In reality, however, transient flow occurs inside the mound due to external wind, diurnal temperature changes and other environmental influences, all of which can significantly affect mound performance. Thermal properties like conductivity and heat capacity are also commonly treated as constant, even though they vary with temperature. Similarly, properties such as permeability, CO₂ diffusivity and thermal conductivity are often assumed to be isotropic. Yet, termite mounds exhibit anisotropy due to their complex, porous and directional structure as shown in figure 6. Further simplifications, such as assuming ideal gas behaviour for CO₂ transport or ignoring turbulence, may also limit the model’s ability to reflect real-world conditions, especially under varying thermal gradients.

Conducting a sensitivity analysis can help assess how these assumptions influence model accuracy and identify which factors most affect performance. Furthermore, the results of simulations should be validated with field or experimental data to ensure the model’s accuracy. For instance, Gouttefarde *et al*. [83] fitted field temperature data from *Procornitermes araujoi* mounds to the heat equation to estimate diffusivity and forcing terms and showed good agreement between the equation and measured data. The heat diffusivity coefficient was also found to be similar to that of soils with comparable properties. Validating the simulations can be challenging in situations where numerical simulations are not accompanied by fieldwork or laboratory experiments. In such cases, the model’s results can be compared with field or experimental data from similar mounds in similar ecological contexts, if available, or benchmarked against other validated numerical simulation results. For instance, Ocko *et al*. [16] compared the optimized morphologies generated by their model to natural termite mound morphologies across different environments.

In the future, flow field simulations could incorporate dynamic mound structures that change over time to produce more realistic results. Additionally, a multiscale modelling approach combining microscale and millimetre-scale simulations should be explored. Geochemical analyses, including mineralogical and elemental composition studies, could also provide deeper insight into mound materials, with these data serving as inputs for more accurate modelling.

## 7. A new proposed integrated approach

Seventy years of research on thermoregulation and ventilation in termite mounds has yielded numerous key insights:

— Termite mounds maintain stable internal temperatures in their nests with fluctuations of only 0–4°C despite fluctuating external conditions [9,26,32–34]. This implies that termite mounds act as natural climate control systems, enabling colonies to thrive in harsh environments.
— Both inhabited and uninhabited mounds exhibit similar temperature stability [32,34–37]. This implies that the mound’s architectural design, rather than termite activity, is the key factor in maintaining internal temperature stability.
— Among mounds of the same species and shape, larger mounds show greater temperature stability than smaller ones [5,32,34] with a smaller coefficient of variation (CV) and range (see table 1). This is attributed to their larger thermal mass which allows them to retain heat for longer periods while resisting thermal fluctuations.
— Mound architecture adapts to environmental changes [1,23,44]. Thus, the architecture of the mound can reflect the environmental conditions in which it is built.
— Termite mounds consist of a porous network of air channels of varying connectivity and sizes [51–53] (see table 2).
— Mound walls contain micropores that aid in insulation, CO_2_ diffusion, permeability, drainage and structural stability [50].
— External winds may influence mound ventilation, especially in regions and periods of high wind activity [9,10,26].
— Diurnal temperature fluctuations influence ventilation within mounds [9,38]. These fluctuations create thermal gradients that drive convective airflow within the mound, facilitating gas exchange with the ambient environment.

There are many outstanding questions related to climate control in termite mounds. Some key questions are:

— What specific roles do structural elements like chambers and galleries play in ventilation and gas regulation? This understanding would allow us to identify mechanisms rather than relying solely on observation.
— Which configurations or mound architectures best support climate control? Is any mound architecture preferred over others?
— Do micropores serve the same function in fungus-farming termites as in non-fungus-farming species, or is their role species-specific?
— How do smaller mounds, lacking chimneys and large air conduits, achieve ventilation and how does this compare with larger mounds?
— How do mounds manage effective ventilation in high-humidity conditions?
— Can insights across species reveal general principles in structure–function relationships?
— What processes occur at smaller scales in the mound that control larger-scale observations?

**Figure 10.**
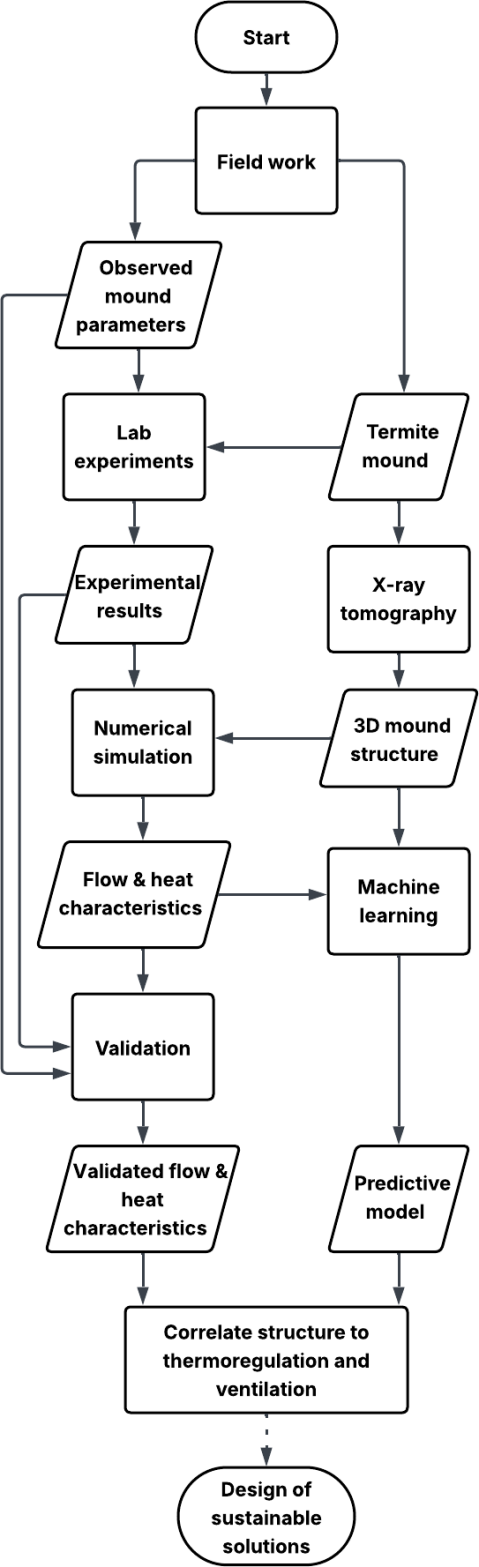
Workflow from termite mound analysis to the design of sustainable solutions. Start/end 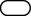, process 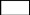, input/output 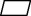. Termite mounds are obtained from field work and imaged in three dimensions using X-ray tomography. Three-dimensional images provide input data for numerical simulations to understand flow characteristics and thermal performance of the mound. Laboratory experiments on mound samples can also be performed providing useful parameters for the numerical simulations. Numerical simulations are then validated using results from laboratory experiments or data collected in the field. Additionally, flow characteristics and three-dimensional mound structure are fed into machine learning to identify structural patterns and correlations, predicting how structure affects thermoregulation and ventilation aiding in the design of sustainable solutions inspired by termite mounds. The broken line between the final two steps indicates that the connection is indirect and may involve multiple additional steps.

Addressing these questions requires an interdisciplinary approach, combining expertise from biology, physics, engineering and other research fields like X-ray tomography, numerical modelling and machine learning. X-ray tomography enables visualizing and analysing mound structures across various scales. The three-dimensional images acquired using X-ray tomography can be used for multiscale numerical modelling to study pressure and velocity fields, permeability, heat and CO_2_ transport within the mound. Controlled laboratory experiments on mound samples can further complement the simulation findings by examining behaviour under varying conditions, including temperature, gas concentrations, humidity and drainage. These experiments would also help validate the results from numerical simulation. Machine learning techniques applied to imaging data and numerical simulation results can develop predictive models that reveal patterns in channel networks and solid arrangements, shedding light on their functional impact. Structural features obtained from the three-dimensional images of the mound can be compared with the flow properties to identify important correlations. This integrated approach, shown in figure 10, will enable a better understanding of the mechanisms that provide effective climate control in termite mounds leading to sustainable solutions.

Implementing these sustainable solutions on a large scale in human architecture and other systems would be a complex and long-term endeavour. This is because local environmental conditions, material variations and human comfort requirements differ significantly from those in termite mounds, and these factors must be integrated with existing technologies. Several additional steps are required after establishing the link between mound structure and climate control to develop sustainable solutions. These steps include design, material selection, prototyping, testing, optimization, scaling and implementation, all of which also involve collaboration across multiple disciplines. In the short term, the primary focus should be on understanding the structural elements that effectively support climate control in termite mounds, which will serve as a basis for achieving long-term sustainability.

## 8. Conclusions

This study highlights the remarkable ability of termite mounds in achieving thermoregulation and ventilation through their complex architectural structures, offering invaluable insights for sustainable building designs and other green solutions. Despite decades of research studying key features, such as the thermal stability of both inhabited and uninhabited mounds across species and ventilation driven by diurnal temperature changes, some fundamental questions remain unanswered. These include the roles of chambers and galleries in airflow, how smaller species achieve ventilation without large conduits and how these mechanisms compare with those in larger mounds. Addressing these questions requires an interdisciplinary approach, combining X-ray tomography and flow field simulations to quantify critical structural and flow properties of the mound.

We have demonstrated how X-ray tomography can reveal the internal geometry of termite mounds at both macroscopic and microscopic scales, capturing essential details such as micropores and channels that facilitate gas exchange and temperature regulation. Coupled with numerical simulations, one can visualize dynamic processes like heat flow, CO_2_ transport and airflow patterns, providing deeper insights into how these structures function under varying environmental conditions. We have also proposed that a multiscale approach combining millimetre-scale and microscale images of the mound should be adopted for realistic modelling of the entire mound. Numerical simulations should be validated using field or experimental data, as inadequate validation limits the reliability and comparability of results across studies.

This study also emphasizes the adaptability of termite mounds, showcasing how structural variations across species and environments are fine-tuned to balance competing demands such as temperature stability, gas exchange and moisture regulation. The ability of termites to achieve such sophisticated climatic control using simple materials and passive mechanisms holds immense potential for biomimicry in sustainable architecture and engineering. Applications range from designing energy-efficient buildings to developing advanced ventilation systems that reduce energy consumption.

In the past, some building designs have been inspired by termite mounds, such as the Eastgate Centre built in 1996 in Harare, Zimbabwe [10], which achieved a 17−52% reduction in energy consumption compared with similar buildings [89]. Other examples include the Davis Alpine House in London, England, which utilizes the stack effect for passive cooling [89], the Nianing Church in Senegal, which finds inspiration in the termite mound model aiming at passive thermal regulation [90], as well as London’s Portcullis House in England and Council House Two (CH2) in Melbourne, Australia [91]. These buildings are referred to as ‘bio-mythological inspired’ [12] because, although they attempt to mimic the processes in termite mounds, they do not fully replicate these processes due to incomplete understanding. It would be interesting to explore the actual energy savings that could be achieved by applying the real principles at work in termite mounds to buildings. This would involve treating the building as a machine [10] and adapting construction techniques to the local environment using local materials [1].

Future research should focus on further refining this integrated approach by incorporating machine learning to analyse patterns in termite mound structures and predict optimal designs for specific environmental contexts. Moreover, other methods, such as microindentation testing and XRD analysis [47,50,92] can be incorporated into this approach to provide a comprehensive understanding of the material properties contributing to climate control in the mound. Statistical methods, such as multiple regression analysis, can also help identify which structural properties have the greatest impact on the mound’s internal climate. Graph theory also provides valuable insights into the connectivity and flow pathways within the mound by modelling its internal structure as a network [93–97]. Additionally, expanding studies across a broader range of termite species and environmental settings will help uncover general principles underlying their climate control strategies. These insights could pave the way for transformative innovations in construction and environmental management, directly inspired by nature’s engineers [98].

By merging biology, physics and engineering, this study sets the stage for exploring the untapped potential of termite mounds as models for sustainable design. It reinforces the idea that understanding the natural world can inspire practical, scalable solutions for pressing human challenges such as energy efficiency and climate resilience.

## Data Availability

The data files used for generating the figures can be found in the Zenodo open‑access repository [99].
